# Supplementary material for: Microscale geometrical modulation of PIEZO1 mediated mechanosensing through cytoskeletal redistribution
Source: Nat Commun. 2024 Jun 29;15:5521. doi: 10.1038/s41467-024-49833-6 (PMC11217425; doi:10.1038/s41467-024-49833-6)
Supplement: Supplementary file 1 — Supplementary Information [file 41467_2024_49833_MOESM1_ESM.pdf]

Title: Microscale geometrical modulation of PIEZO1 mediated mechanosensing through cytoskeletal redistribution

Authors: Haoqing Jerry Wang[1,2,3], Yao Wang[1], Seyed Sajad Mirjavadi[4], Tomas Andersen[1], Laura Moldovan[1,2,3], ParhamVatankhah[1], Blake Russell[1], Jasmine Jin[1], Zijing Zhou[5], Qing Li[4], Charles D. Cox[5,6], Qian Peter Su[3,7*], Lining Arnold Ju[1,2,3,8*]

[1] *School of Biomedical Engineering, The University of Sydney, Darlington, NSW 2008, Australia*

[2] *Charles Perkins Centre, The University of Sydney, Camperdown, NSW 2006, Australia*

[3] *Heart Research Institute, Camperdown, Newtown, NSW 2042, Australia*

[4] *School of Aerospace, Mechanical and Mechatronic Engineering, The University of Sydney, Darlington, NSW 2008, Australia*

[5] *Molecular Cardiology and Biophysics Division, Victor Chang Cardiac Research Institute, Sydney, NSW 2010, Australia*

[6] *Faculty of Medicine, St. Vincent's Clinical School, University of New South Wale, Sydney, NSW 2010, Australia*

[7] *School of Biomedical Engineering, University of Technology Sydney, Sydney, NSW 2007, Australia*

[8] *The University of Sydney Nano Institute (Sydney Nano), The University of Sydney, Camperdown, NSW 2006, Australia*

** Correspondence: arnold.ju@sydney.edu.au, qian.su@uts.edu.au*

The supporting material contains:

1. Supplementary Figures S1-S14
2. Supplementary Tables
3. Supplementary Method

1. Supplementary Figures

Figure S1 Micropipette orifice diameter and tip angle distribution

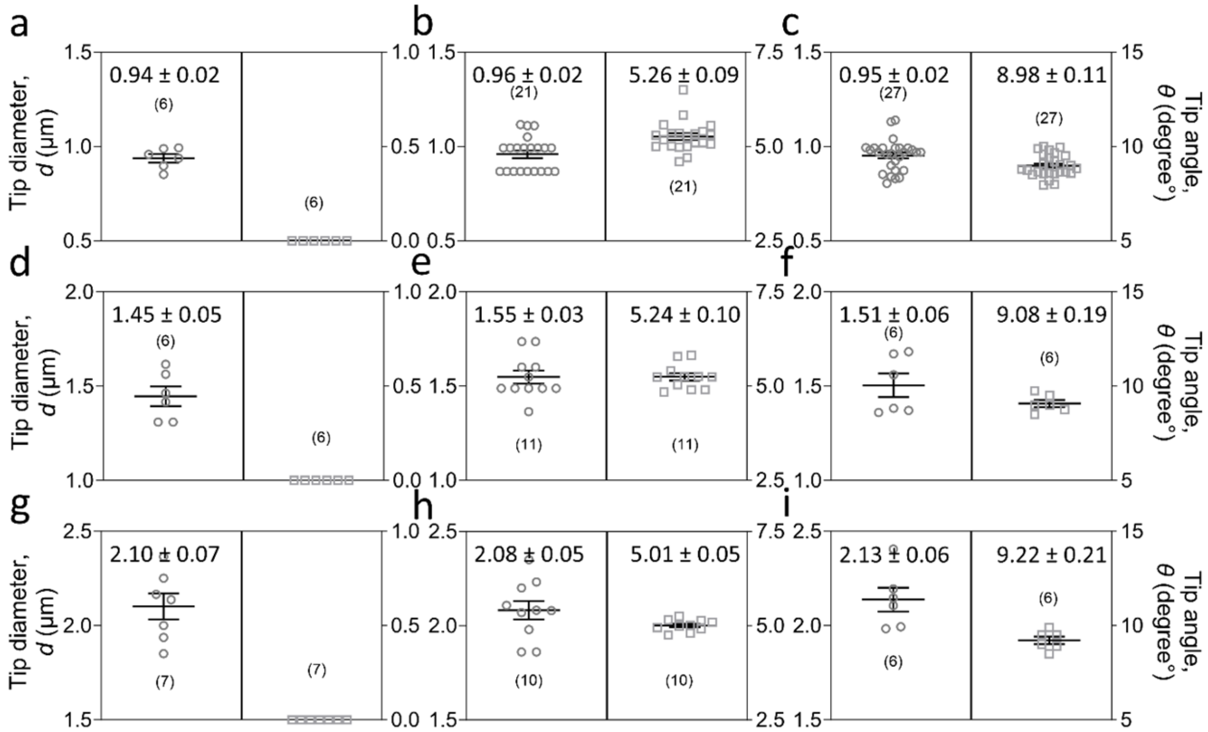

The distribution of micropipette tip angle (*right*) distribution in this study when tip diameter (*left*) was set to be 1 µm (**a-c**), 1.5 µm (**d-f**), and 2 µm (**g-i**), respectively. Number of measure micropipettes is labeled, and the data is represented as mean ± s.e.m.

Figure S2 Drug validation on PIEZO1-mediated Ca^{2+} influx.

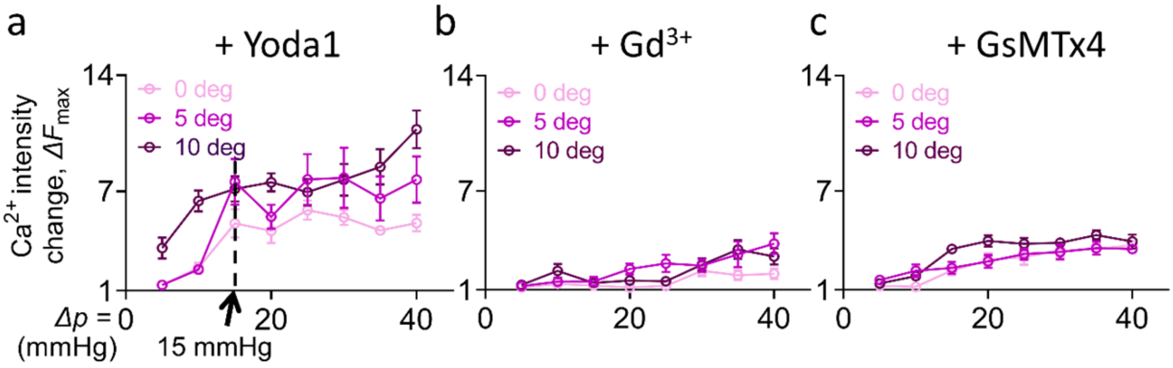

a Piezo1 agonist sensitized RBC Ca^{2+}-dependent mechanosensing. ΔF_{max}–Δp curve was plotted over the pressure range Δp = -5 to -40 mmHg (n = 72 at θ = 0°; n = 83 at θ = 5°; n = 139 at θ = 10°). 0.5 µM Yoda1 was flushed into the RBC cell solution before fMPA. Ca^{2+} intensity at low-pressure range was enhanced (-15mmHg, *arrow pointed*), indicating Yoda1 induced a much lower gating threshold of the Ca^{2+} influx. **b** Ca^{2+} influx is mediated by ion channels. 50 µM Gd^{3+} blocking suppressed the Ca^{2+} ΔF_{max} across all pressure, indicating the ΔF_{max} is due to the Ca^{2+} influx via the ion channels (n = 88 at θ = 0°; n = 86 at θ = 5°; n = 74 at θ = 10°). **c** The ion channel is the membrane tension dependant channel, PIEZO1. After incubation with 2.5 µM GsMTx4, the Ca^{2+} influx was suppressed by relieved membrane tension (n = 89 at θ = 0°; n = 123 at θ = 5°; n = 101 at θ = 10°). All data are presented as mean ± s.e.m.

Figure S3 ***Piezo1*-KO^{RBC} suppressed calcium mobile zation in aspirated RBCs**

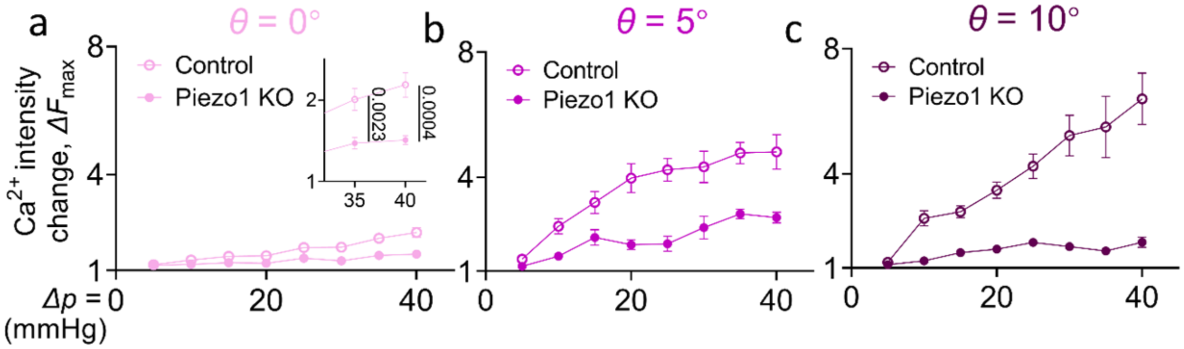

Control (EpoR-Cre) and *Piezo1*-KO^{RBC} mouse whole blood was washed to isolated RBCs. fMPA assay was then performed with $\theta = 0°$ (**a**; WT: $n = 123$, KO: $n = 123$), 5° (**b**; WT: $n = 110$, KO: $n = 97$), and 10° (**c**; WT: $n = 175$, KO: $n = 169$) micropipette. Ca^{2+} ΔF_{max} exhibited a similar trend over the aspiration pressure range in WT mouse RBC (*open circle*) compared to human RBC, while the response was inhibited in the *Piezo1*-KO^{RBC} (*solid circle*). Data are presented as mean ± s.e.m., assessed by Welch *t*-test.

Figure S4 Aspirated cell body-to-tongue ratio at all aspiration pressure (corresponding to Fig. 2h)

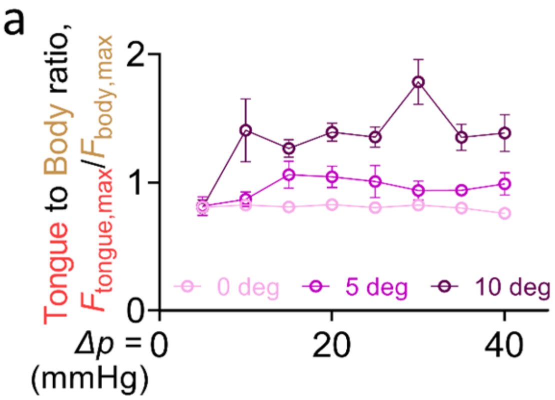

RBC was aspirated by $\theta = 0°$ (*pink*), 5° (*magenta*) and 10° (*purple*) micropipette at aspiration pressure Δp = -5 to -40 mmHg (n = 202 at $\theta = 0°$; n = 84 at $\theta = 5°$; n = 100 at $\theta = 10°$). The difference was not significant when Δp = -5 mmHg, implying the aspiration-induced membrane tension change was comparable across all tip angles. However, a noticeable difference was observed across tip angles when the aspiration pressure increased to -15 mmHg and the trend remains the same across the rest of the pressure range. were aspirated under every condition to obtain mean ± s.e.m.

Figure S5 Pearson's correlation test between FLIM vs. fMPA and FLIM vs. FEA

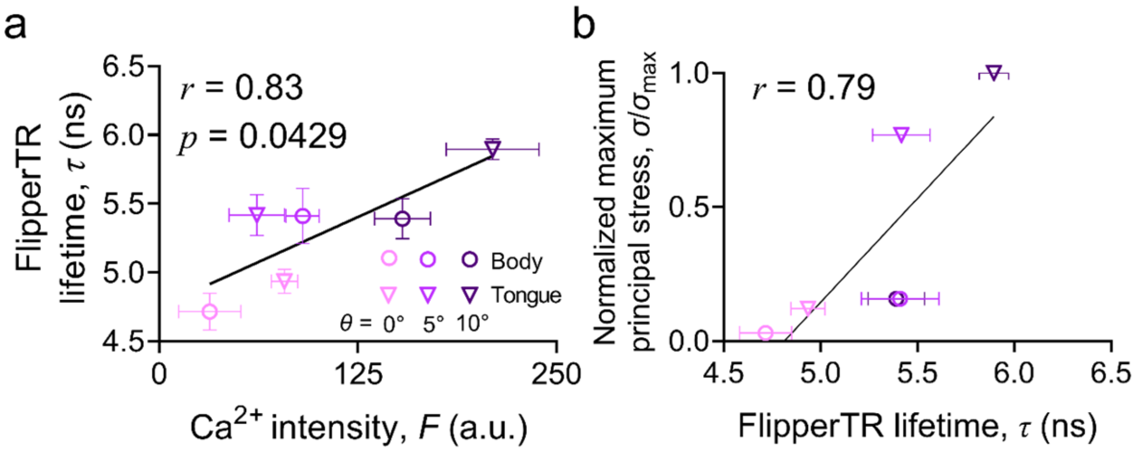

Evaluation of results correlation. Pearson correlation tests was performed between FLIM versus fMPA (**a**) and FEA versus FLIM (**b**) results at Δp = -25mmHg. Statistical outcome demonstrated a strong positive correlation between FLIM versus fMPA results (r = 0.83) and FEA versus FLIM results (r = 0.79). The correlation underscores our hypothesis that tip angle variation is a key mechanical factor influencing PIEZO1 channel activity, further supported by FLIM data and FEA results demonstrating the tension increase with larger tip angles.

Figure S6 High local membrane tension induces dumbbell morphology in aspirated RBC

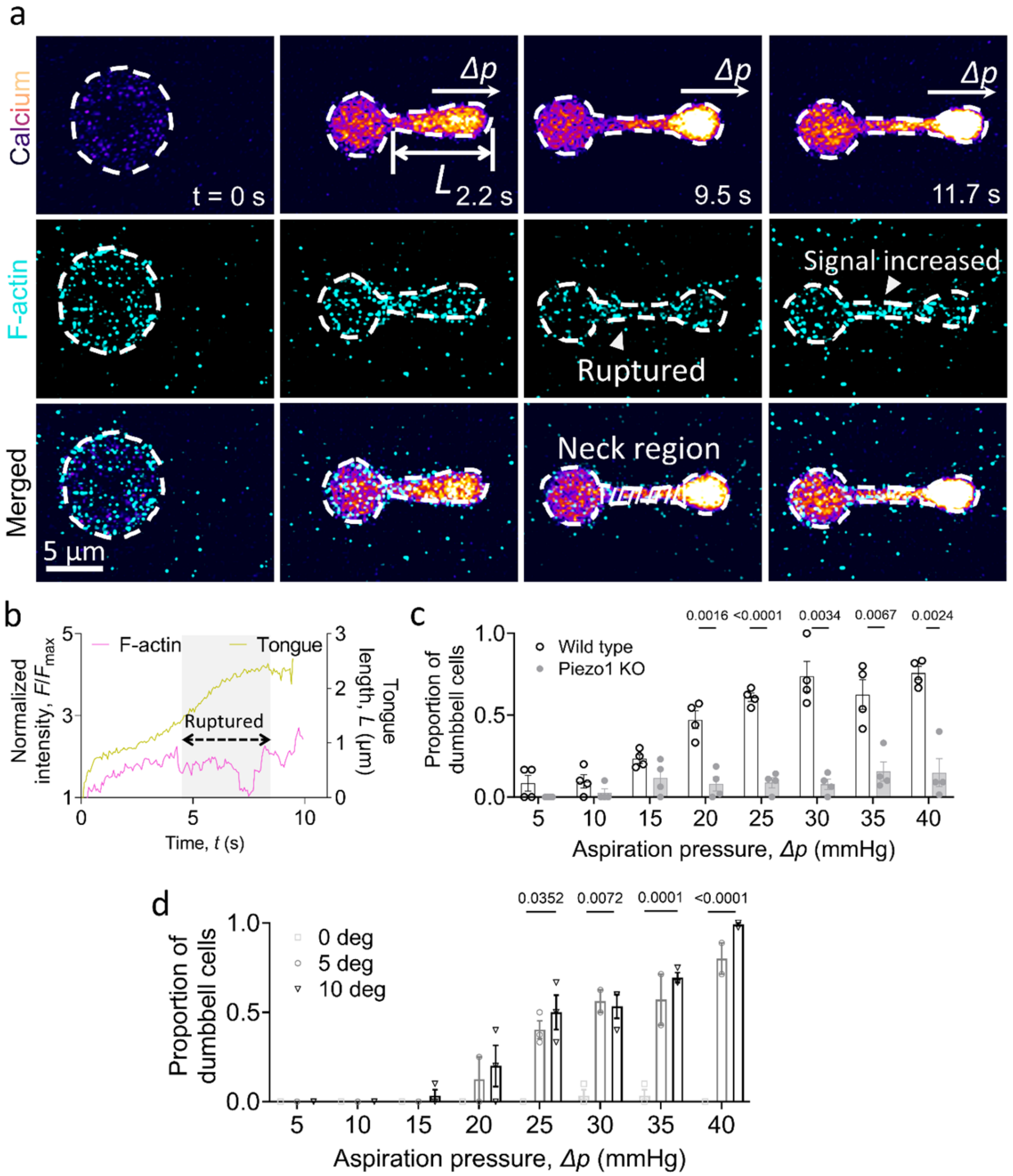

a Representative snapshot of RBC being aspirated by a $\theta = 10°$ micropipette. The calcium (*top*), F-actin (*middle*) were concurrently captured on the FV3000 confocal microscope. **b** When the cell was aspirated by Δp = -40 mmHg, a dumbbell morphology was formed which is characterized by a sudden increase in the tongue length, L. The F-actin rupture was indicated by a sudden decrease of the signal during the tongue length increase. **c** The proportion of bottlenecked cells is PIEZO1 level dependant. When *Piezo1*-KO^{RBC} was aspirated by the θ = 10° micropipette, a limited number of cells exhibited dumbbell morphology at all pressure. n = 4 mice samples for each phenotype were tested to obtain mean ± s.e.m, comparison was assessed by Welch's t-test. **d** The dumbbell morphology is mechanical force dependent. Higher

tip angles and higher aspiration pressure induced an increase in dumbbell morphological cells. $n = 3$ mice samples for each phenotype were tested to obtain mean ± s.e.m, comparison was assessed by Welch's *t*-test.

Figure S7 Calcium influx in HEK293T cells is PIEZO1 mediated (corresponding to Fig. 4b).

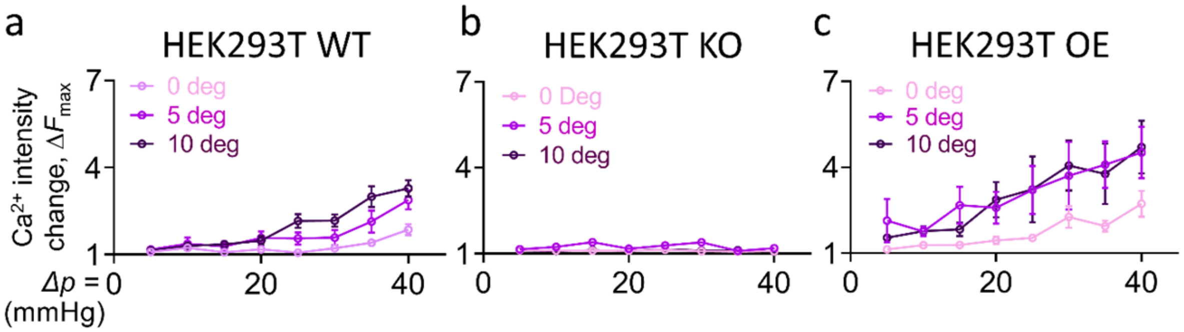

fMPA aspiration assay was performed on WT (**a**; $n = 81$ at $\theta = 0°$; $n = 45$ at $\theta = 5°$; $n = 108$ at $\theta = 10°$), PIEZO1-KO (**b**; $n = 52$ at $\theta = 0°$; $n = 50$ at $\theta = 5°$; $n = 53$ at $\theta = 10°$), and PIEZO1-OE (**c**; $n = 59$ at $\theta = 0°$; $n = 58$ at $\theta = 5°$; $n = 51$ at $\theta = 10°$) HEK293T cells respectively. Calcium influx was completely inhibited when PIEZO1 was eliminated in HEK293T cells[1]. In sharp contrast, the response was upregulated by the PIEZO1 expression level. These results demonstrated the calcium influx in the HEK293T cell during fMPA assay was PIEZO1 mediated. Data are presented as mean ± s.e.m., assessed by Welch *t*-test.

Figure S8 F-actin accumulation is mediated by aspiration force and PIEZO1 expression level dependent.

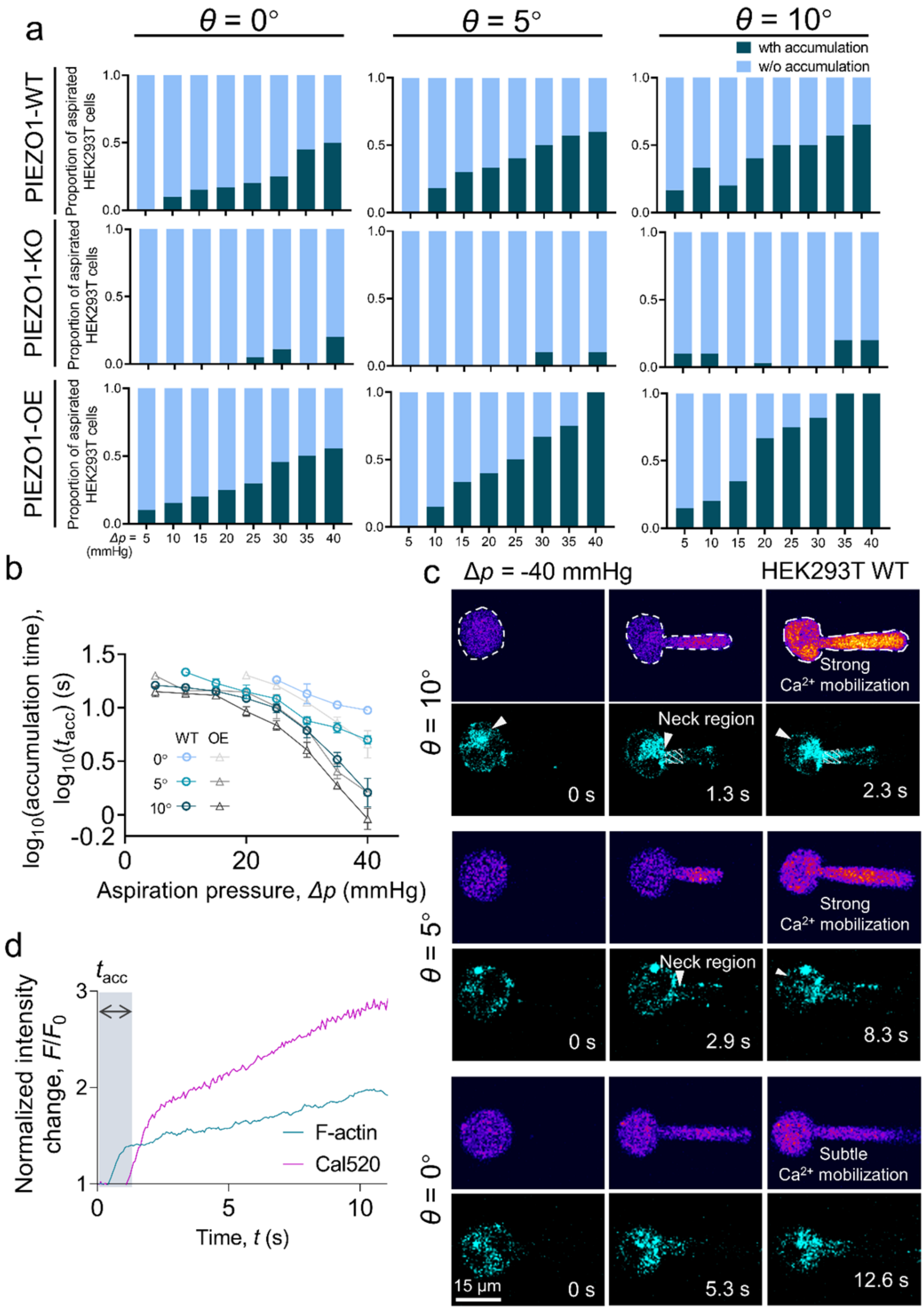

a Full data set of the proportion of aspirated Wild type, PIEZO1-KO, and PIEZO1-OE HEK293Tcells with F-actin accumulation (corresponding to Figs. 5b-d). **b** Full data set of the F-actin accumulation time of aspirated WT (n = 7 at $\theta = 0°$; n = 29 at $\theta = 5°$; n = 29 at $\theta = 10°$)

and Piezo1-OE (n = 17 at θ = 0°; n = 27 at θ = 5°; n = 30 at θ = 10°) HEK293T (corresponding Fig. 5e). All data are shown in mean ± s.e.m. **c** Representative snapshot of HEK293T WT cell aspirated by θ = 0° (*bottom*), 5° (*middle*), and 10° (*top*) micropipette. The Ca^{2+} and F-actin signal was imaged concurrently while the whole cell Ca^{2+} intensity and F-actin signal inside the neck region was quantified over time. **d** Representative trace of F-actin signal inside the neck region and Ca^{2+} signal of the whole cell over the aspiration process. F-actin accumulation time was measured from the start of aspiration until the F-actin signal in the neck region increased more than 1.5-fold and stabilized for more than 2 seconds. It is noted that a Ca^{2+}intensity rapid increase occurred after F-actin accumulation, indicating that F-actin played a vital role in strong Ca^{2+} mobilization.

Figure S9 Full Ca^{2+} ΔF_{max}–Δp curve of fMPA aspiration on F-actin restricted HEK293T WT cells (corresponding to Fig. 5f)

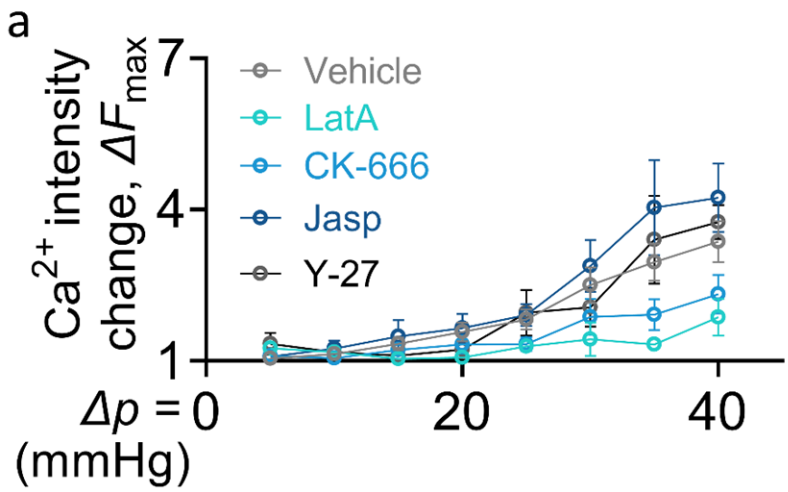

HEK293T WT cells were treated with 1:500 DMSO (Vehicle, n = 113), 100 µM CK-666 (n = 66), 2.5 µM Latrunculin A (LatA, n = 56), 10 µM Jasplakinolide (Jasp, n = 54), and 50 µM Y-27632 (Y-27, n = 55), respectively, before fMPA was performed. LatA and CK-666 treatment significantly suppressed Ca^{2+} mobilization compared to vehicle treatment. In contrast, Jasp stabilized the F-actin structure and further amplified the Ca^{2+} influx in the aspirated cells at high pressure. F-actin contractility inhibition had a limited effect, indicating that the role of F-actin in PIEZO1 activity amplification is independent of cytoskeleton contractility. All data are shown in mean ± s.e.m.

Figure S10 Full Ca^{2+} ΔF_{max}–Δp curve of fMPA aspiration on adhered and suspended HEK293T WT (corresponding to Fig. 6h)

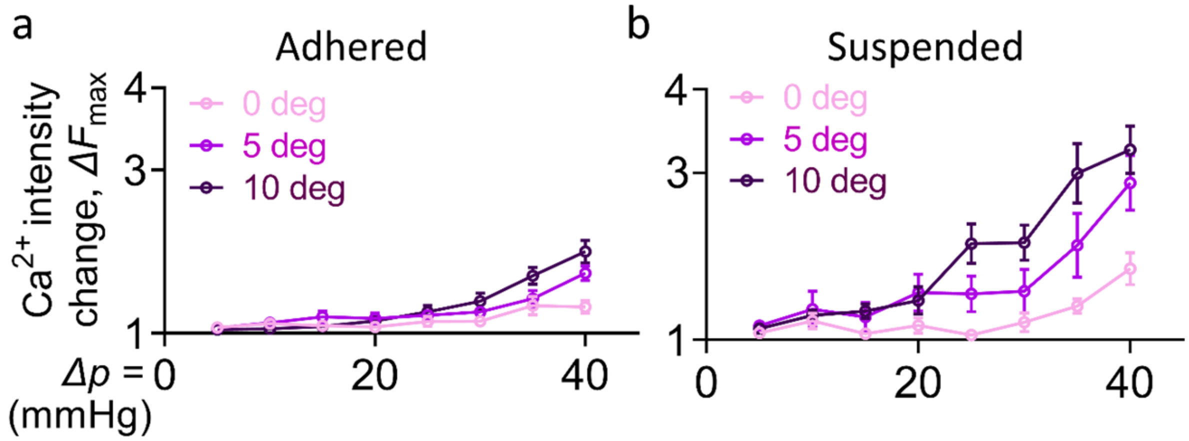

HEK293T cells were aspirated when they were attached to an FN-coated cover glass (**a**; n = 48 at $\theta = 0°$; $n = 48$ at $\theta = 5°$; $n = 48$ at $\theta = 10°$) or suspended in the Tyrode's buffer (**b**; $n = 80$ at $\theta = 0°$; $n = 45$ at $\theta = 5°$; $n = 110$ at $\theta = 10°$). Lower Ca^{2+} ΔF_{max} was noticed for adhered cells, indicating that restriction of F-actin movement towards the micropipette tip would prevent the PIEZO1 activity amplification. All data are shown in mean ± s.e.m.

Figure S11 PIEZO1/F-actin co-location in hP1-mCherry HEK293T aspirated by θ = 0°, 5° and 10° (corresponding to Fig. 7e)

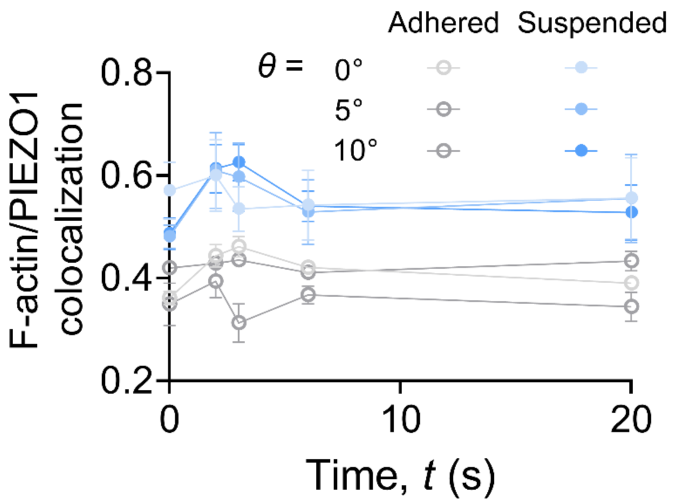

PIEZO and F-actin co-localization was quantified for adhered and suspended cell during micropipette aspiration. Results were analyzed from n = 8 cell from two independent experiments. All data are shown in mean ± s.e.m.

Figure S12 Schematic of distinct membrane curvatures around PIEZO1 in RBC (a; smooth and flat) and HEK293T (b; invaginated), respectively.

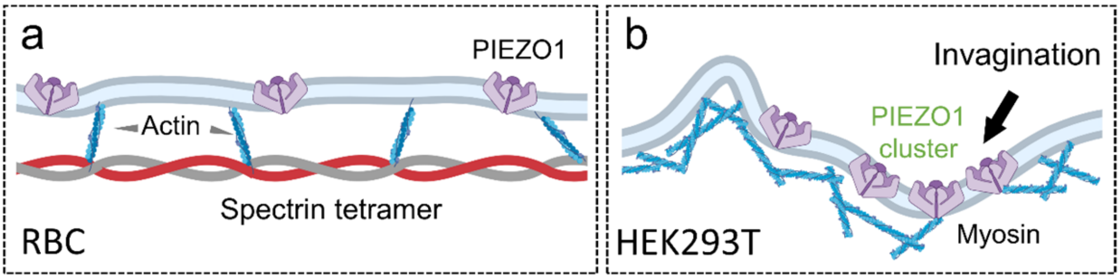

Figure is created with BioRender.com released under a Creative Commons Attribution-NonCommercial-NoDerivs 4.0 International license https://creativecommons.org/licenses/by-nc-nd/4.0/deed.en

Figure S13 Schematic of multi-stage micropipette pulling

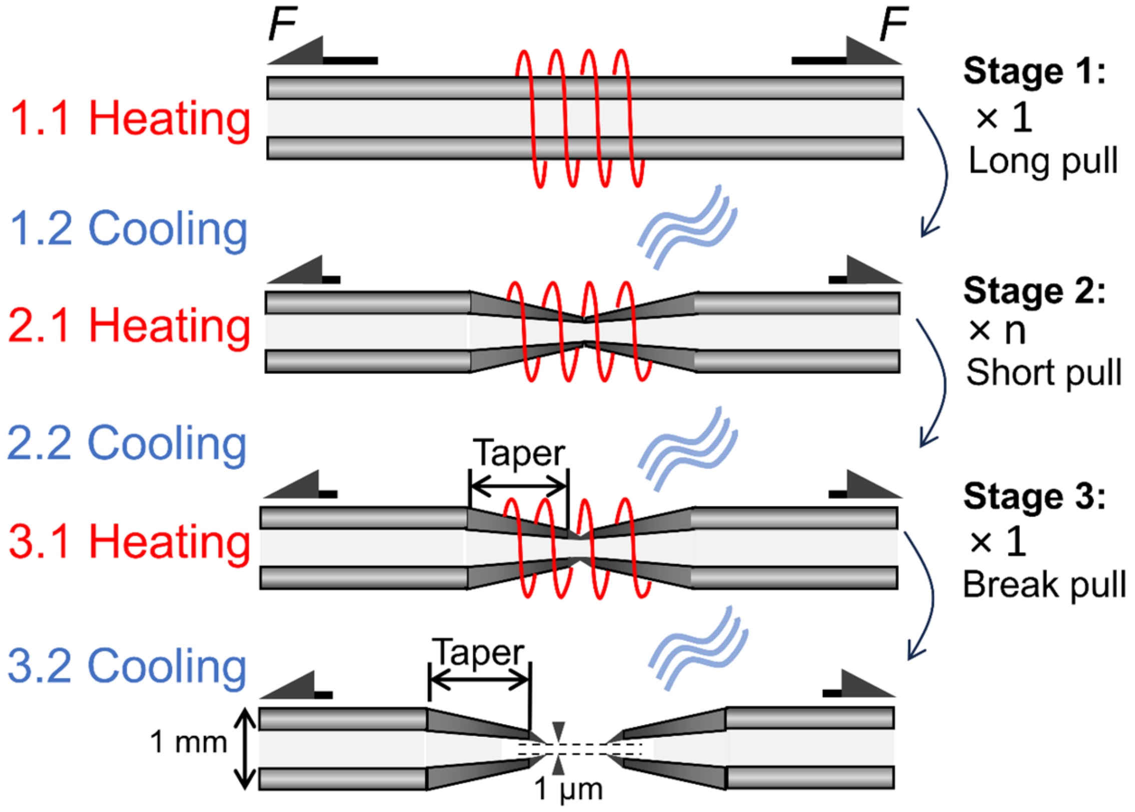

To pull an angled opening micropipette with enough taper length, the glass capillary was mounted on the micropipette puller to perform multi-stage pulling. A constant heat (termed 'Heat') was applied by the filament in the middle (*red*) to turn glass into fluid while the pulley at either end would apply a constant force (*F*) to slowly pull the capillary apart. When the sensor detected the moving velocity of the pulley (termed 'Velocity'), the heating would stop, and a strong pressure (termed 'Pressure') airflow was facilitated to cool the glass down. In Stage 1, a long pull was utilized to obtain a long taper in the fabricated micropipette, achieved by a pre-set Velocity at a high value to prevent breakage of the glass. After cooling down, several short pulls (based on the requirement) were applied in Stage 2 (i.e., low Velocity) to finely fabricate the micropipette into detailed geometries. Lastly, a slightly longer pull was applied in Stage 3 to give a clean cut between fabricated micropipettes. Detailed parameters are listed in Supplementary Table 1.

Figure S14 Modelling red blood cell deformation upon micropipette aspiration.

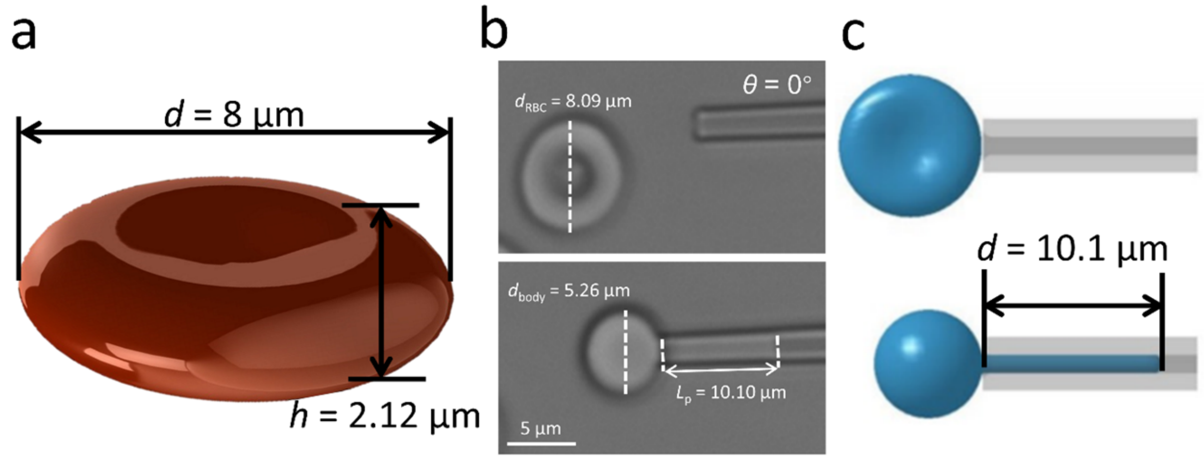

a Initial model setup for the RBC aspiration modeling, displaying the undeformed biconcave geometry of a real RBC as used in the computational simulations**.** The undeformed state serves as a baseline for subsequent deformation analysis. The model was generated with SOLIDWORKS 2021 and is available in Source Data Files. **b** Snapshots of RBC aspirated by $\theta = 0°$. Measurement was performed using ImageJ after cell mask segmentation. **c** Elastic shell finite element analysis (FEA) modeling schematic. The figure was generated in LS-PrePost . Biconcave shaped RBC was placed next to the micropipette first, representing the initial stage. Then aspirated RBC dimension was reconstructed and meshed. The model in LS-DYNA is available in the Source Data Files.

2. Supplementary Table 1

Material list

Name	Source	Catalog Number
Cal-520 AM	Abcam	ab171868
Yoda1	Cayman Chemical Company	21904
Latrunculin A	Abcam	ab144290
CK-666	Sigma-Aldrich	182515
Jasplakinolide	Life Technologies	J7473
Y-27632	Sapphire Bioscience	S1049
SPY650-FastAct	Spirochrome	SC505
Flipper-TR	Spirochrome	SC020
DMSO, Anhydrous	Thermo Fisher Scientific	D12345
GsMTx4	Abcam	ab141871
Gadolinium(III) chloride	Sigma-Aldrich	439770
RPMI Medium 1640	Thermo Fisher Scientific	22400-089
Fetal Bovine Serum	Thermo Fisher Scientific	26140-079
TrypLE^{TM} Express	Thermo Fisher Scientific	12604-021
Human Fibronectin	Thermo Fisher Scientific	33016-015
Penicillin/Streptomycin	Thermo Fisher Scientific	15140122
DPBS	Thermo Fisher Scientific	14040133
Sodium bicarbonate	Sigma-Aldrich	S9638
HEPES	Sigma-Aldrich	H3375
Sodium carbonate	Sigma-Aldrich	S2127
D-(+)-Glucose	Sigma-Aldrich	G7021
Sodium chloride	Sigma-Aldrich	S7653
Potassium chloride	Sigma-Aldrich	P9541

3. Supplementary method

3.1 Extracting the contour of FEA simulation.

To extract the contour of stress after FEA simulation, the outcome was post-processed in the LS-PrePost V4.10.8 (2020). The 1st principal stress was chosen to illustrate the membrane tension along the cell surface after aspiration. The customized fringe range was set to demonstrate the stress distribution normalized by the maximum stress magnitude amongst the results compared, **Supplementary Table 2**. A customized palette file was implemented to adjust the figure's colormap (**Figs. 3f-h and Figs. 5g-h**).

In order to covert the rainbow color palette in LS-Dyna to Batlow format, we implement the following steps:

1) After going to LS-PrePost V4.5.24 (2020), we will go to the Fringe range, we choose "user radio button" option and then load "Batlow Fringe color palette" and then put the Min and Max based on the Supplementary Table 2.
2) the level should be set to 30 in the same window.

Supplementary Table 2:Parameters for LS-PrePost figure output

Fig	Min	Max	Division by
3f-h	0	2.8e-01	2.8e-01
5g and 5h	1.991e-09	4e-04	4e-04

References

1 Dubin, A. E. *et al.* Endogenous Piezo1 Can Confound Mechanically Activated Channel Identification and Characterization. *Neuron* **94**, 266-270 e263 (2017). https://doi.org:10.1016/j.neuron.2017.03.039
