# Supplementary material for: Microscale geometrical modulation of PIEZO1 mediated mechanosensing through cytoskeletal redistribution
Source: Nat Commun. 2024 Jun 29;15:5521. doi: 10.1038/s41467-024-49833-6 (PMC11217425; doi:10.1038/s41467-024-49833-6)
Supplement: Supplementary file 3 — Reporting Summary [file 41467_2024_49833_MOESM3_ESM.pdf]

nature portfolio

Corresponding author(s): Lining Arnold Ju, Qian Peter Su

Last updated by author(s): 11/03/2024

Reporting Summary

Nature Portfolio wishes to improve the reproducibility of the work that we publish. This form provides structure for consistency and transparency in reporting. For further information on Nature Portfolio policies, see our Editorial Policies and the Editorial Policy Checklist.

Statistics

For all statistical analyses, confirm that the following items are present in the figure legend, table legend, main text, or Methods section.

n/a	Confirmed	
☐	☒	The exact sample size (*n*) for each experimental group/condition, given as a discrete number and unit of measurement
☐	☒	A statement on whether measurements were taken from distinct samples or whether the same sample was measured repeatedly
☐	☒	The statistical test(s) used AND whether they are one- or two-sided<br>*Only common tests should be described solely by name; describe more complex techniques in the Methods section.*
☒	☐	A description of all covariates tested
☐	☒	A description of any assumptions or corrections, such as tests of normality and adjustment for multiple comparisons
☐	☒	A full description of the statistical parameters including central tendency (e.g. means) or other basic estimates (e.g. regression coefficient) AND variation (e.g. standard deviation) or associated estimates of uncertainty (e.g. confidence intervals)
☐	☒	For null hypothesis testing, the test statistic (e.g. *F*, *t*, *r*) with confidence intervals, effect sizes, degrees of freedom and *P* value noted<br>*Give P values as exact values whenever suitable.*
☒	☐	For Bayesian analysis, information on the choice of priors and Markov chain Monte Carlo settings
☒	☐	For hierarchical and complex designs, identification of the appropriate level for tests and full reporting of outcomes
☐	☒	Estimates of effect sizes (e.g. Cohen's *d*, Pearson's *r*), indicating how they were calculated

Our web collection on statistics for biologists contains articles on many of the points above.

Software and code

Policy information about availability of computer code

Data collection	Micro-Manager Version 2.0.0 was used to collect 2-channels concurrent imaging. Micropipette aspiration system was controlled by LabVIEW 2019 (National Instrument). Olympus FV31S-SW was used to perform imaging on the confocal microscope. Cell-attached patch clamping data was collected with AxoScope 10.7 (Molecular Devices). Finite element analysis was conducted using ANSYS LS-DYNA R13.1.0 (ANSYS) and LS-Run2023 (ANSYS). Initial model was built in SOLIDWORKS 2020 (Dassault Systèmes). FLIM data were collected using SymPhoTime 64 software (Picoquant). Spinning disk confocal imaging utilized Slidebook capture software (Intelligent Imaging Innovations) for acquisition.
Data analysis	GraphPad Prism 9 was used for data plotting and statistical analysis. All fluorescence image was quantitatively analyzed by ImageJ 1.53 (Fiji, National Institutes of Health) and Imaris 9.0.1 (Oxford Instruments). Finite element analysis post-processing was conducted using LS-PrePost V4.10.8. Patch clamping data was analyzed by using pCLAMP10 software (Molecular Devices). FLIM data was analyzed with FLIMfit 5.1.1.

For manuscripts utilizing custom algorithms or software that are central to the research but not yet described in published literature, software must be made available to editors and reviewers. We strongly encourage code deposition in a community repository (e.g. GitHub). See the Nature Portfolio guidelines for submitting code & software for further information.

Data

Policy information about availability of data

All manuscripts must include a data availability statement. This statement should provide the following information, where applicable:

- Accession codes, unique identifiers, or web links for publicly available datasets
- A description of any restrictions on data availability
- For clinical datasets or third party data, please ensure that the statement adheres to our policy

The authors declare that the data supporting the findings of this study are available within the paper and its supplementary information files. The relevant raw data from each figure are provided in the Source data file. Source data are provided with this paper.

Research involving human participants, their data, or biological material

Policy information about studies with human participants or human data. See also policy information about sex, gender (identity/presentation), and sexual orientation and race, ethnicity and racism.

Reporting on sex and gender	This study recruited healthy subjects of both genders.
Reporting on race, ethnicity, or other socially relevant groupings	This study recruited healthy subjects of different ages (18-60) and varied races (Caucasian, Asian, African American).
Population characteristics	Subjects with no bleeding-related disorders (e.g. von Willebrand's Disease) and cardiovascular diseases histories, who are not currently using anti-blood clotting or anti-inflammatory medication, including nonprescription items such as aspirin or ibuprofen (e.g. Nurofen™, Heron Blue™) in past 3 weeks, were chosen for the study. No more than 400 mL of blood, which is the amount of blood that is taken by the Blood Bank during a routine blood donation, was collected from any given volunteer per visit.
Recruitment	After the notice was sent out, the first volunteer that responded was always chosen as the blood donor. No bias of selection can be envisioned. All participants and their accompanying guardians were provided with a verbal explanation of the study. A verbal agreement and a signed consent form were respectively collected from the participants and accompanying guardians, stating that they agree for blood donation, and for the publication of the data collected using their blood.
Ethics oversight	All experiments were performed in accordance with relevant guidelines and approved by the University of Sydney Human Research Ethics Committee (HREC, project 2023/582).

Note that full information on the approval of the study protocol must also be provided in the manuscript.

Field-specific reporting

Please select the one below that is the best fit for your research. If you are not sure, read the appropriate sections before making your selection.

☒ Life sciences ☐ Behavioural & social sciences ☐ Ecological, evolutionary & environmental sciences

For a reference copy of the document with all sections, see nature.com/documents/nr-reporting-summary-flat.pdf

Life sciences study design

All studies must disclose on these points even when the disclosure is negative.

Sample size	Sample size were chosen based on our previous publication (Wang et al., 2022, PMID: 35286429) and other studies in this field, which provide significant statistical information and data reproducibility. The exact sample size (such as the number of cells) are mentioned in figure legends and methods. No sample size calculation was performed.
Data exclusions	For fluorescence quantification analysis, cells ruptured during aspiration were excluded from analysis.
Replication	The experimental findings were reproduced in multiple independent experiments on different days. The number of biological replicates for each data panel is indicated in the figure panel itself and methods.
Randomization	No formal randomization techniques were used; however, samples were allocated randomly to experiments and processed in an arbitrary order.
Blinding	The investigators were blinded to group allocation during data collection and analysis where possible. Experiment involving human red blood cells, investigators were blinded to the identity of blood donors. Experiments involving red blood cell-specific PIEZO1 knockout mice were analyzed in a manner blinded to treatment identity. After collecting the data, the samples were re-identified so the results could be interpreted.

Reporting for specific materials, systems and methods

We require information from authors about some types of materials, experimental systems and methods used in many studies. Here, indicate whether each material, system or method listed is relevant to your study. If you are not sure if a list item applies to your research, read the appropriate section before selecting a response.

Materials & experimental systems

n/a	Involved in the study
☒	☐ Antibodies
☐	☒ Eukaryotic cell lines
☒	☐ Palaeontology and archaeology
☐	☒ Animals and other organisms
☒	☐ Clinical data
☒	☐ Dual use research of concern
☒	☐ Plants

Methods

n/a	Involved in the study
☒	☐ ChIP-seq
☒	☐ Flow cytometry
☒	☐ MRI-based neuroimaging

Eukaryotic cell lines

Policy information about cell lines and Sex and Gender in Research

Cell line source(s)	HEK293T wildtype were purchased from Thermo Fisher Scientific. HEK293T PIEZO1 knockout was from Dr Charles D. Cox and Dr Boris Martinac labs, and originally and kindly gifted from Dr Ardem Patapoutian Lab. HEK293T PIEZO1 overexpressed was from Dr Charles D. Cox and Dr Boris Martinac labs, and originally and kindly gifted from Dr Philip Gottlieb.
Authentication	HEK293T wildtype was purchased from the distribution. PIEZO1 knockout and overexpressed cell lines were validated via calcium fluorescent imaging after Yoda1 (PIEZO1-targeted agonist) treatment. No further authentication has been introduced in this study.
Mycoplasma contamination	The cell line was tested negative for mycoplasma contamination.
Commonly misidentified lines (See ICLAC register)	No commonly misidentified cell line was used in the study.

Animals and other research organisms

Policy information about studies involving animals; ARRIVE guidelines recommended for reporting animal research, and Sex and Gender in Research

Laboratory animals	Six to eight weeks old Piezo1-KO/RBC and Piezo1/flox mice were used. We generated RBC-specific PIEZO1 knockout mice (Piezo1-KORBC) with erythropoietin receptor (EpoR) Cre recombinase. Subsequently, we introduced the Piezo1flox alleles to the EpoR-Cre mice. The first generation of this crossbreeding resulted in all traits being heterozygous. The next step involved back-crossing these heterozygous mice with homozygous Piezo1flox animals to produce the Piezo1-KO_RBC experimental subjects, where the EpoR-Cre induced recombination specifically in the erythroid lineage, knocking out the Piezo1 gene. All male mice with C57BL/6J background and carrying and EpoR-Cre were bred and sourced from Australian BioResources (ABR; Moss Vale, NSW, Australia). Piezo1flox mice were obtained from Jackson Laboratories (JAX stock 029213). The mice were maintained in a light/dark cycle of 12 h/12 h, at a temperature of 21 °C and 50% humidity.
Wild animals	No wild animal were used
Reporting on sex	Sex was not considered in study design.
Field-collected samples	No wild animals were used.
Ethics oversight	All experimental procedures were approved by the Animal Ethics Committee of Garvan/St Vincent's (Australia), in accordance with the guidelines of both the Australian Code for the care and use of animals for scientific purposes (8th edition, National Health and Medical Research Council, Australia, 2013).

Note that full information on the approval of the study protocol must also be provided in the manuscript.
